# Book Review

**Published:** 1942

**Authors:** 


### Reviews of Books

**The Medical Annual, 1941.** Edited by H. Letheby Tidy, M.D., F.R.C.P., and A. Rendle Short, M.D., F.R.C.S. Pp. lvi., 516. Illustrated. Bristol : John Wright & Sons Ltd. 1941. Price 23s.—In spite of grave difficulties the *Medical Annual* was published in 1941 about the usual date : a performance on which the publishers deserve the highest praise. This volume contains several contributions of exceptional interest, particularly those relating to pharmacology. One of these discusses the advances made in the group of sulphonamides, but lays insufficient stress on the dangers of granulocytopenia and the absolute necessity for blood counts during the administration of sulphonamides. The other deals with addenda to the B.P. and with some new barbiturates. Here *epanutin* is severely criticized, its relationship to the discredited drug *nirvanol* is pointed out and the writer of the article doubts whether *epanutin* has any real place in medicine. Epilepsy and electro-encephalography form the subject of two articles. One is a survey of those conditions in which electro-encephalography has proved most useful. In the other a critical enquiry is made into the methods of various observers. The view is expressed that the different standards employed by different workers constitute a real weakness, and it is regretted that before the norms have been determined electro-encephalography should be regarded as capable of dealing with pathological states. The article on Blood Transfusion is of very great value. In Surgery one of the most interesting articles is that on Head Injuries, showing that these casualties in the present war have a much better chance of surviving than in the war 1914–18. Peptic ulcer is considered both medically and surgically at length and the whole survey is admirable. One might go on picking out many other articles of exceptional interest, but it is unnecessary after fifty-nine years of successful production to do more than to say that Wright's *Annual* is as good as ever.

**Common Skin Diseases.** Sixth Edition. By A. C. Roxburgh, M.D., F.R.C.P. Pp. xx., 436. Illustrated. London : H. K. Lewis & Co. Ltd. 1941. Price 16s.—The writing of text-books for medical students on special subjects has become, with the advance of knowledge, an increasingly difficult task, since the time allotted for the student on such subjects, in the already overburdened curriculum, is very short. Dr. A. C. Roxburgh has overcome this difficulty with much success and has contrived to accentuate those points which are essential. Both text and figures show evidence of full revision, such as in the section on Monilia and Impetigo, whilst other sections show expansion, as in the section on Industrial Dermatitis. The up-to-date character of this new edition is shown in the section on Thorinum X, Dermatomyositis and

Congenital Syphilis. The book abounds in useful advice based on well-proven methods of diagnosis and treatment. It is compact, clearly written and freely illustrated. It is a great achievement, for which author and publisher must be congratulated, to have produced a book like this in its Sixth Edition despite of the trials of the past two years.

**Massage and Remedial Exercises.** Fifth Edition. By N. M. TIDY. Pp. xii., 463. Illustrated. Bristol: John Wright & Sons Ltd. 1941. Price 17s. 6d.—It is scarcely two years since we reviewed the fourth edition of this veritable encyclopædia of remedial exercises, essential reference-book for senior students, and teachers of massage and medical gymnastics. Very little has been altered, but some useful notes on amputation stumps, also on burns and wounds have been added. The illustrations on page 256 seem to have been transposed. We hope, when more propitious days come along, that a really new edition will appear, with larger, less crowded type, also more and not quite such old-fashioned illustrations.

**The Treatment of Burns.** By A. B. WALLACE, M.Sc., F.R.C.S. Pp. xiii., 113. Illustrated. London: Oxford University Press (Humphrey Milford). 1941. Price 5s.—Mr. Wallace has written a timely and interesting little book on the treatment of burns, a subject which is at present being much debated. In his chapter on classification he gives one most valuable sub-division of burns into those suitable for out-patient treatment and those which must be admitted to hospital. His advice on first aid is definite and noteworthy: "To apply any substance locally does not materially help the patient and certainly creates difficulties for the surgeon." Now this advice, if it represents the general opinion of surgeons, makes revision of all First Aid Manuals urgently necessary. Henceforward the first aid treatment can be expressed very briefly "leave the burn alone and treat the patient" unless the burn is exposed when it may be covered with a sterile dressing or a clean cloth. The patient is to be kept warm and given fluids to drink. His descriptions of the various ways of treating major burns are adequate, but he is not quite definite in expressing preference for selecting any particular form of treatment. An inexperienced reader might justifiably feel a good deal of doubt in choosing a method. In his discussion of the use of picric acid the author mentions that untoward results from picric acid are recorded in literature but that he has never seen any untoward sequelæ. This, to the present reviewer, suggests some limitation of experience, since severe reactions to picric acid are not by any means rare. The author's advice on the treatment of initial and secondary shock is well thought out and the details are easy to follow: although it is not very helpful to be told that in administering oxygen for secondary shock the best results are obtained by intermittent applications of high concentrations according to the patient's need. We are given no indication at all as to the methods of judging when to stop or when to continue the administration of oxygen. We are told a flow-meter should be employed, but we are nowhere told to keep the patient's colour pink, which, after all, is the supreme guide. None the less, this is an extremely valuable book for the careful details it contains. It is an excellent reference book for those with sufficient experience to use their own judgment in the choice of treatments. It only just fails to be a wholly satisfactory counsellor to the inexperienced.

**Practical Orthoptics in the Treatment of Squint.** Second Edition. By KEITH LYLE, M.D., F.R.C.S., and SYLVIA JACKSON. Pp. xv., 341. Illustrated. London: H. K. Lewis & Co. Ltd. 1940. Price 15s.—Originally intended for the instruction of orthoptic students, and written by authors with much experience in the subject, this book has become well known as an excellent practical manual on squint-training. In the second edition, its scope and size have been increased by the inclusion of new chapters and much additional information. Besides the sections on orthoptic instruments and methods of treatment, there are chapters on the etiology of squint (based on original work by the late Bernard Chavasse), on adult cases of strabismus, on heterophoria and paralytic squint. In recent years, an increasing amount of attention has been given to ocular torticollis and its treatment, a subject which is here fully discussed. To the ophthalmologist, these special chapters with descriptions of illustrative cases, along with the appendix, will probably prove the most interesting part of the book. The appendix contains an analysis of over 500 cases investigated and treated during twelve months at the Royal Westminster Ophthalmic Hospital, and gives some idea of the results to be expected in different types of squint. Orthoptic treatment has now developed into an important branch of ophthalmic work, and is generally regarded as a valuable (if not indispensable) method in treating certain types of strabismus and ocular muscle-imbalance. This book can be recommended with confidence to all concerned with the problems of squint as a lucid, interesting and up-to-date presentation of the subject.

**First Aid to the Injured and Sick.** Eighteenth Edition. By WARWICK and TUNSTALL. Edited by NORMAN HAMMER, M.R.C.S. Pp. vi., 336. Illustrated. Bristol: John Wright & Sons Ltd. 1941. Price 3s. 6d.—Just forty years have elapsed since this excellent handbook was first published. For some years the new editions had been edited by our late colleague Mr. F. C. Nichols, whose recent death we so deeply deplore. This latest (eighteenth) edition comes from the hands of Major Norman Hammer. His knowledge of A.R.P. and Civil Defence work in general has given him singular qualifications for his task, and he has successfully revitalized the book. A great deal of parade drill has been wisely omitted and replaced by practical hints on first aid in general and special advice on the collection and immediate care of air-raid casualties. The seventeenth edition was prepared before the outbreak of war in 1939, the eighteenth has been written with full knowledge of the bitter lessons of the past two years and the handling of military and civilian casualties. The editor is to be congratulated on the able way he has incorporated his new material in this time-honoured manual.

**Fourth Addendum to the British Pharmacopœia, 1932.** Pp. viii., 32. London: General Medical Council. 1941.—This book, like its predecessor, presents two main features, both arising from the particular difficulties of wartime Materia Medica. The first is the inclusion of a number of drugs and preparations representing official equivalents for preparations hitherto known under proprietary titles, *e.g.* Injectio Mersalyl (B.P.) = Salyrgan, Pamaquin = Plasmoquin, Phenylhydrargyri Nitras = Merfenil, Suramin = Germanin. Secondly, the inclusion of a number of drugs, now frequently, prescribed, but not hitherto officially recognised., *e.g.* Acid Nicotinic, Benzyl Benzoate, Bismuth Subgallate, Magnesium Trisilicate, Proflavine Sulphate, Sulphanilamide. The Benzyl Benzoate is now an essential ingredient in the modern treatment of scabies, and sulphonamides may be regarded as overdue for B.P. inclusion. Perhaps Appendix XVI is the most important innovation, dealing with sterilisation processes for parenteral injections. The always unsatisfactory methods of Tyndallisation and Emergency Sterilisation are deleted, and replaced by heating with a Bactericide such as Chlorocresol or Phenyl Mercuric Nitrate. A list of solutions follows with their appropriate methods of sterilisation.

**Dental Materia Medica, Pharmacology and Therapeutics.** Second Edition. By W. J. DILLING, M.B., Ch.B., and S. HALLAM, L.D.S., R.C.S. Pp. xvi., 348. London: Cassell and Co. Ltd. 1941. Price 13s. 6d.—The first edition of this text-book has already been warmly accepted by lecturers and dental students. This second edition has been brought thoroughly up to date and includes a very helpful résumé of (1) The Sulphonamides, (2) Antiseptic Dyes, (3) Recent and buffered Local Anæsthetics, (4) The Analeptics, and (5) a number of other recent therapeutic agents. The section on Vitamins, followed by a short section on the Ductless Gland Hormones influencing metabolism, is comprehensive and should prove invaluable to the student. Specific reference is made to premedication—in view of its increasing use in dental surgery a helpful table of the drugs in this group is given. The new terminology adopted by the British Pharmacopœia Commission for drugs formerly described under the registered trade names has been adopted. This, at first sight, may cause some confusion especially to lecturers. The section on filling materials has been considerably curtailed, reference only being made to those of a temporary nature and for root canal therapy. The book, taken as a whole, reads well and tries to avoid the tedium of listed drugs. It is a change to find a text-book with sections instead of chapters—these sections are so arranged they lead conveniently on from one group of drugs to another. While general pharmacology is clearly expressed, concentration on the dental side, with special reference to the oral cavity, is kept prominently to the forefront. This book should be placed in the front rank of text-books on the subject.

